# Prosocial Involvement as a Positive Youth Development Construct: A Conceptual Review

**DOI:** 10.1100/2012/769158

**Published:** 2012-04-30

**Authors:** Ching Man Lam

**Affiliations:** Department of Social Work, The Chinese University of Hong Kong, Hong Kong

## Abstract

This paper discusses the concept of prosocial involvement as a positive youth development construct. How prosocial involvement is defined and how the different theories conceptualize prosocial involvement are reviewed. Antecedents of prosocial involvement such as biological traits, personality, cognitive and emotional processes, socialization experience, culture, and their social context are examined. The relationship between prosocial involvement and adolescent developmental outcomes, together with strategies to promote prosocial involvement in adolescents, are discussed. Finally, directions for future research and practice are proposed.

## 1. Introduction

Prosocial involvement, namely, the tendency for people to act voluntarily to benefit others has been regarded as a basic tenet of human nature and is also a cardinal virtue of all societies. A number of studies attest to the positive influence that prosocial involvement exerts on individual functioning and interpersonal transactions. In the various positive youth development programmes, prosocial involvement opportunities and activities are significant and important elements for the healthy development of adolescents [1]. The involvement of adolescents in prosocial activities serves the functions of making adolescents aware of and able to accept the social norms and moral standards of society. This involvement will bring positive changes to the adolescents and consequently benefit society as a whole. In Hong Kong, professionals in education and social services have fully recognized the benefits of prosocial involvement activities for youth, and already there are diverse prosocial involvement programmes organized for adolescents' participation. This paper reviews the concept and theories of prosocial involvement, and how prosocial involvement behaviour can be promoted in the local context to enhance the healthy development of adolescents.

## 2. Definition of the Construct

The terms “helping behaviour,” “prosocial behaviour,” and “altruism” are frequently used interchangeably when discussing the construct of prosocial involvement. Although these terms are closely interrelated, they may be distinguished from each other for analytic purposes. 

According to Bierhoff [2], “helping behaviour” is the broadest term, including all forms of interpersonal support, whereas the meaning of prosocial behaviour is narrower in that the action is intended to improve the situation of the help-recipient. Prosocial behaviour usually refers to voluntary actions that are intended to help or benefit another individual or group of individuals [3]. Prosocial behaviour is defined in terms of consequences intended for another in which the behaviour of the actor is directed toward promoting and sustaining a positive benefit for the help-recipient. Also, the behaviour is performed voluntarily rather than under duress and is not motivated by the fulfilment of professional obligation. Activities such as donating, sharing, helping, assisting, and providing support to someone else are regarded as prosocial behaviour, whereas paid activities in the service sector are usually excluded [2]. Altruism is one specific type of prosocial behaviour. It refers to voluntary acts that are intended to benefit others and are intrinsically motivated, that is, acts motivated by internal motives such as concern, sympathy, or by altruistic values [2]. Altruism is characterised by an emphasis on the needs of the others, concern about their wellbeing, and finding a solution for their problems. There is no expectation of receiving a reward in any form except perhaps a feeling of having done a good deed. Besides, altruism includes a belief in the importance of the welfare and just treatment of others, and is characterised by taking perspective and by empathy [3]. Therefore, what determines whether or not a prosocial action is considered altruistic is the motive underlying the behaviour—it is the intention of the helper that determines an altruistic act, and motivation is what distinguishes more general prosocial behaviour from altruism.

A closely related concept is prosocial norms. Prosocial norms refer to the clear and healthy ethical standards, beliefs, and behavioural guidelines that promote prosocial behaviour and minimize health hazards [4]. Prosocial norms are defined as standards and clear beliefs which point to the shared expectations of behaviours in society that are considered healthy, ethical, culturally desirable, and appropriate [5]. These shared expectations are very often formalized and constitute a control mechanism of society in that one is expected to act in accordance with learned or internalized norms.

Despite the differentiation between general helping behaviour and altruism in the literature, altruistic behaviour is not distinguished from more general prosocial behaviour when referring to prosocial involvement. Prosocial involvement refers to events or activities across different settings that an individual or group of individuals participate in, with the express purpose of benefitting others. Prosocial and altruistic behaviours are all regarded as prosocial involvement manifesting in forms such as purposive or accidental helping, sharing, donating, comforting, and servicing and vary depending on motivation and the degree of self-sacrifice involved on the part of the actor.

## 3. Assessment of Prosocial Involvement

Prosocial involvement can be assessed using a quantitative approach, a qualitative approach, or even a mixed approach to triangulate the data. In the quantitative approach, scales are used to assess prosocial involvement. For example, “The Self-Report Altruism Scale” [6] is a 20-item Likert-type scale that assesses the frequency with which participants engage in behaviours such as volunteer work or helping strangers in a particular situation. The Prosocial Orientation Questionnaire [7] contains 40 statements on a 4-point scale that measures various aspects of the prosocial orientation and behaviour of adolescents. Other instruments include the “Prosocial Behaviour Scale” [8], the “Prosocial Self-regulation Questionnaire,” [9] and the “Prosocial Reasoning Objective Measure” [10]. Self-rating, peer-rating, teacher-rating, and/or parent-rating are the most frequently used methods of assessment.

Another method frequently used to assess prosocial involvement is the observational method. Observational studies of children's pro-antisocial behaviour have been reported regularly since the 1930s [11]. This kind of study usually applied the time sampling procedure, which means that during a predetermined time interval the occurrence or nonoccurrence of specified pro-antisocial behaviours is scored. Naturalistic observations [3] focus on children's behaviour in their “natural” environments such as playgrounds, homes, or classrooms, whereas “situational tests” involve controlled settings designed to elicit prosocial responses (i.e., requiring the children to play games or perform a task). The frequency of occurrence of a response in a given time period will be recorded systematically. An alternative is the summary evaluation by raters in which observers retrospectively rated categories of behaviour over a fixed period of observation. A third method is that a reference person, that is, a parent or teacher gives a rating of the child's prosocial behaviour.

Qualitative methods employ open-ended questions, drawing, reflective logs, and case studies to examine prosocial involvement. For example, adolescents are invited to discuss their experiences in community involvement or to reflect on their expectations, motives and goals in their prosocial involvement. Using a hypothetic scenario and asking participants for their response is a frequently used method for studying individual and cultural differences in prosocial responses. One of the first studies of this kind dealt with the willingness of a target person to mail a stamped letter for a stranger who made the request at a train station [12].

## 4. Theories of Prosocial Involvement

There are different theoretical explanations for the development of prosocial dispositions. According to psychoanalytic theory [13], there are three major structures of personality, namely, the id, the ego, and the superego. The one that is most relevant to an understanding of prosocial involvement is the superego. The superego reflects the standards of society and sets a person's moral standards or ideals. The role of superego in the process of personality development is of considerable importance as this is the process for individuals to internalize humanistic values and patterns of prosocial involvement.

Social learning theories maintain that most human behaviour is learned, moulded, and shaped by environmental events, especially rewards, punishments, and modelling. From the perspective of social learning, prosocial involvement is interpreted as the consequent of reinforcement or punishment. Social approval encourages prosocial involvement, whereas social disapproval is expected to lead to a reduction in the targeted behaviour. Study results [14] clearly indicate that children's behaviour with respect to sharing possessions or helping someone in distress will be strengthened if it results in them being rewarded by praise or attention. Another study also confirmed that approval or disapproval of the model behaviour provided a cognitive script for modelling [15]. Principles of conditioning and learning have been used to explain the development of empathy and a tendency toward altruism.

Building on social learning theory, social cognition theorists propose that humans act on the environment just as the environment acts on them. According to Bandura [16], there are self-evaluation processes that set internal standards and rules for behaviour. Individuals set goals for their behaviour, anticipate the outcome of their behaviour, and then act in ways that bring that desired outcome. Therefore moral development, including prosocial involvement, is a product of the interaction between socialization and the individual's cognition.

Theoretical approaches also highlighted the role of motivation in prosocial involvement. According to a functional analysis of altruism [17], prosocial involvement satisfies the needs or motives of the individual. Motivational functions such as the expression of values, social responsibility or career enrichment enhance prosocial involvement [17]. Wentzel et al.'s study [18] indicates that goal pursuit significantly predicted prosocial involvement, and goal pursuit provided a pathway to relate reasons for behaviour to actual behaviour. Their studies identify a range of self-processes that motivate displays of prosocial involvement. Theoretical perspectives on motivation have been used to explain the development of prosocial involvement, and prosocial involvement is conceptualized as the outcome of self-processes that satisfy individual goals.

The different theoretical conceptualizations of prosocial involvement reveal several major mechanisms in the learning of prosocial involvement-prosocial modelling, social reinforcement, moral internalization/self-processing, and altruistic attributes. Modelling is a social process through which behaviour patterns are acquired and transmitted. It involves observational learning, identification, and imitation [16]. Usually adults and significant others such as teachers or peers may act as prosocial models for children and adolescents. Social reinforcement is based on either reward or punishment and the role of social reinforcement for the facilitation or inhabitation of prosocial involvement has been demonstrated in studies [19]. A self-process involves reasons for behaviour and perspective taking relates to prosocial involvement. Finally, altruistic attributes such as altruistic personality and altruistic self-concept may function as an internalized standard of prosocial involvement, that is, activated across a broad spectrum of social situations.

## 5. Antecedents of Prosocial Involvement

Many factors, including biological, personal, interpersonal and cultural, are antecedents of prosocial involvement. The question is whether people are altruistic by nature or by nurture? Evolutionary theorists and geneticists have long understood how certain physical traits are genetically determined, and biological factors no doubt play a role in the capacity for prosocial involvement. MacLean's [20] review of research suggests that the brain activities related to prosocial involvement. Many twin studies have also found that there are genetic bases for the predisposition to act altruistically [21].

At the personal level, personal or personality variables are factors related to prosocial involvement. Gender, age, social class, and personality traits are the most frequently mentioned individual characteristics that are associated with prosocial involvement. Although there is no clear and consistent evidence of gender difference in prosocial responses, girls may perform some types of prosocial behaviour more frequently than boys. Personality traits such as assertiveness, gregariousness, and sociability are positively associated with prosocial involvement [22]. Penner's [23] description of the volunteer process included the prosocial personality as an antecedent to sustained helping. His other study reveals that there are traits that comprise “prosocial personality” [24] and there are significant associations between these clusters of prosocial dispositions and prosocial action [23]. These findings suggest that there are personality trails that form a prosocial personality.

Cognitive processes, which refer to the actor's perception, interpretation, and evaluation of a situation, are another important determinant. Research on the development of prosocial involvement has identified a range of cognitive processes likely to motivate displays of prosocial actions including the level of cognitive development, perspective taking, and the level of moral reasoning [25]. Self-efficacy beliefs and self-transcendence values (i.e., benevolence and universalism) operate in concert to promote prosocial involvement [26].

The development of prosocial involvement involves an emotional process of empathy. Studies revealed a theoretical and empirical association between empathy [27] or sympathy [28] and children's prosocial involvement. Theories of altruistic and prosocial involvement assert that prosocial behaviour is enacted empathy [29], and empathy is a mechanism by which people's altruistic nature is expressed. Research indicates that feeling empathy for a person in need is an important motivator when it comes to helping [30]. There is research which provides evidence that individual differences in empathy are related to individual differences in prosocial and altruistic behaviour through adolescence and into early adulthood [25, 31]. Regarding the relationship between parenting, empathy, and prosocial involvement, the results of a longitudinal study revealed that warm parenting fosters and models sympathy (empathy) and is a unique predictor of adolescents' prosocial involvement [32].

Socialization experience is another important determinant for prosocial involvement at the interpersonal level. According to socialization theorists [16], parents play an important role in promoting and fostering prosocial involvement in their children and in adolescents. Eisenberg's study [33] reveals that warm parenting facilitates higher levels and other-oriented forms of prosocial moral reasoning. There are studies that indicate that warm parent-child relationships facilitate emotional sensitivity, perspective taking, and prosocial involvement [34, 35]. Hastings et al. [36] found that an authoritative parenting style was associated with more prosocial behaviour two years later. Studies also indicate that parental socialization practices are important contributors to prosocial involvement. Parent's values, discipline, and affection are also related to their children's altruistic behaviour [37]. A longitudinal study showed that parental warmth, sympathy, and prosocial moral reasoning were predictive of prosocial involvement, and early prosocial behaviour predicted later adolescent's prosocial involvement and later parenting [32]. Other socialization agents such as peers, teachers, and the mass media are also critical in the development of prosocial predispositions in children and adolescents. The study also indicates that adolescent's perceptions of teachers' and peers' expectations for prosocial involvement and their perceived threats of punishment related to prosocial goal pursuit as well as to reasons for behaving prosocially [19].

With regard to the cultural factor, it is generally accepted that an individual's actions, motives, orientations, and values are governed by their culture. A comparison of volunteerism in different nations has revealed that long-term involvement in prosocial activities varies widely between different countries. A study by Carlo and his team members [38] reveals cross-national variations in prosocial reasoning. Their other study [33] indicates that cultural norms exert a significant influence on moral reasoning, and moral reasoning is mediated by different socialisation practices that affect motives of involvement. Besides, the societal norm, which is the set of expectations of how one ought to behave, is important for the development of prosocial involvement. Prosocial involvement is being valued in cultures with a high social responsibility norm, that is, cultures where people act on behalf of others, not for material gain or social approval, but for their own self-approval and for the self-administered reward that arises from doing what is right [33].

Lastly, there are the situation conditions and the social context. External factors such as the school environment, the circumstances confronting the individual, and the presence or absence of opportunities may all explain much of prosocial involvement. For example, the occurrence of crisis or calamities such as the Sichuan earthquake in China and the 9/11 attack in the United States arouses peoples' emotional processes of empathy and the genetic drive for helping. It also pushes people out of their comfort zones and encourages them to involve themselves in helping and volunteering activities. There is a body of research indicating that there are individual differences in prosocial responses in specific settings, or at particular points in time, and that prosocial moral behaviour shifted from situation to situation [24].

## 6. Prosocial Involvement and Adolescent Developmental Outcomes

A number of findings attest to the positive influence that prosocial involvement exerts on individual functioning and interpersonal transactions. On the individual level, findings from developmental research show that prosocial involvement is positively correlated with psychosocial adjustment in children and adolescents [39]. Studies also indicated that children with prosocial reputations tended to be high in constructive social skills and attentional regulation and low in negative emotionality [40]. Early prosocial involvement contributes to children's accomplishments in social and academic domains [41, 42]. There are evidences that prosocial involvement promotes integration in the community, enhance positive mood and help individuals to stay healthy and have better life satisfaction. Study results indicate that prosocial involvement serves as a protective factor that fosters self-enhancement, self-acceptance, and successful psychosocial adaptation [43].

Evidence from research supports the idea that prosocial involvement affects the wellbeing of an individual. There is evidence that engagement in prosocial behaviour can foster the basic psychological needs for competence, relatedness and autonomy [44]. Studies on the mental health of volunteers demonstrate that volunteers are less prone to depression [45, 46], they experience greater happiness [47], have greater life satisfaction [48] and self-esteem [49], and also have a lower level of the feeling of hopelessness and are better adjusted to life [44]. Adolescents who participated in prosocial involvement programmes tend to have positive self-perception, more social skills, and increased prosocial attitudes, values, and identities [50]. Other studies indicate that prosocial behaviour is clearly important through the entire lifespan in promoting mutual acceptance and support, and in keeping positive relations among people [51]. There is consistent evidence that prosocial involvement has positive developmental impacts.

The relationship between prosocial involvement and adolescent development outcomes has also been studied by investigating the relationship between prosocial involvement and adolescent problem behaviour. Hirshi [52] indicated that involvement in legitimate activities inhibits deviance because active participation in these activities consumes time. Several cross-sectional and longitudinal studies provide evidence that high school students who engage in prosocial community service activities are less likely to smoke marijuana, abuse alcohol, perform poorly in school, become pregnant, or commit delinquent acts [53]. There is consistent evidence that prosocial involvement not only reduces crime and delinquency, but also serves a rehabilitation and correctional function in delinquent youths [54]. Thus, it is suggested that prosocial involvement be included in discussions about possible solutions to crime, drug use, offender treatment, or exprisoners returning to society.

The long-term effect of prosocial involvement is evident in studies. Results from a longitudinal study on the relations between parenting styles and prosocial involvement provides supportive evidence that early prosocial involvement predicts maternal warmth later on and has an effect on parenting and prosocial development [32]. This finding is consistent with a prior study that engagement in prosocial activities earlier in life facilitates prosocial development later in life [55]. Study findings showed that youth who frequently act prosocially might be prone to develop prosocial traits that might strengthen their moral sense of self [56]. Other findings also show the positive effect that behaving prosocially has across all stages of adult life, [47] and that there is substantial continuity in prosocial involvement from adolescence through the transition to adulthood [57].

## 7. Promotion of Prosocial Involvement in Adolescents

Given the importance of prosocial involvement, it is essential to promote prosocial involvement among young people in order to achieve positive youth development. Since the family and school are important environments for adolescents, it is suggested that home and school be the context in which adolescent prosocial involvement is promoted.

Individual differences in prosocial involvement are partly due to the degree to which children and adolescents internalize the prosocial values and norms of their society. Parents may have a direct influence on the prosocial values and behaviour of children and adolescents. McLellan and Youniss [21] found that parents who volunteer have children who volunteer, and Michalik's [58] study demonstrated that parenting practices and children's sympathetic responses are related to prosocial involvement. Parenting thus is very important and the role of parents should be addressed in prosocial involvement programs. Providing increased opportunities for children and adolescents to witness the direct modelling effects of their parents, have parents influencing prosocial responses in children and adolescents, and the promotion of prosocial norms by parents helps to foster adolescent prosocial involvement.

For the school environment, the first and most important scheme is the cultivation of prosocial involvement as a kind of school culture. School culture refers to the character of the school. It reflects the pattern of values, beliefs, and traditions of the school and is an important contextual variable influencing prosocial involvement. The school environment can influence the students' involvement in prosocial activities if the school promotes the concepts of connectedness and cooperation. The culture is one in which teachers and students care about and support one another and share values, norms, goals, and a sense of belonging. Besides, school can encourage students to participate in and influence group decisions in order to build a sense of community among the students and to develop a normative value of helping cooperative learning strategies. The school culture that stimulates active student participation and promotes other-oriented values that lead to transcending one's self-interest to benefit others will be decisive in promoting prosocial involvement.

Study results indicate that goal pursuit predicts prosocial involvement [18]. People pursue goals they value and goals provide the reference system that sets and guides personal concerns and behaviour. Therefore, school culture must help adolescents to set goals and find ways to achieve goal fulfilment in prosocial involvement. Moreover, people should be encouraged to search for meanings from within their prosocial involvement experiences and to reflect actively on those experiences. These are also ways to promote prosocial motivation and self-endorsed long-term prosocial involvement.

Teacher and peer influence are another significant determinant of adolescent prosocial behaviour in school. Teachers and teacher support were found to act as a positive indicator of adolescents' sense of social responsibility [59]. Thus, teacher support and guidance are a source of encouragement for students involved in school-based prosocial activities. Peer learning, model interpersonal behaviour, and mutual reinforcement are the keys to enhancing prosocial norms and involvement in school.

The presence or absence of opportunities explains much of the extent of prosocial involvement. The school can be the context in which various opportunities for student prosocial involvement are provided. In fact, most Hong Kong schools have already launched noncurriculum-based programs such as a mentor scheme, social service groups, uniform groups, self-initiated social service programs, or joint school service programs. Curricular-based programs such as service learning programs or training programs implemented in the schools may be a key mechanism through which adolescents can experience prosocial involvement. These programs expose students to civic participation and provide participatory opportunities, especially to those who are least likely to participate because of their lack of connections to other institutionalized programs. It may also provide an experience with great potential for change for those who initially have low civic orientations. Besides, with a planned curriculum, students can be taught systematically about the theories and perspective of volunteerism, the meaning of help, and the importance of civic responsibility. Components such as perspective taking, reflective learning, personal growth, and development can be included to benefit both the helpers and the service recipients.

## 8. Future Research and Practice Directions

Research findings indicate that adolescents nowadays are different from previous generations. Howe and Strauss's study of the Millennial generation [60] reveals that the new generation is not empathetic. Konrath's study [61] reported a sharp decline in empathy among college students and there are research findings showing that narcissism levels among university students have gradually increased over the past 25 years.

However, a contradictory picture has been presented by other researchers. According to Sax [62], adolescents' interest in volunteerism has steadily increased since 1990. Kiesa and colleagues [63] surveyed 12 colleges and revealed that Millennials are actually more engaged in the community than their parent's generation. A recent report entitled Canada Survey of Giving, Volunteering and Participating (CSGVP) revealed that 46% of the population aged 15 and over volunteered in the year of 2007 and the highest rates of volunteering were found among young Canadians [64]. Volunteerism among American college students has reached a high record; with intended participation in community service being 30.8% [65]. According to a recent report of the Agency for Volunteer Service, the average man-hours Hong Kong people spent on volunteering increased from 34.8% in 2001 to 87.4% in 2009 [66].

These figures inform us of further research and practice directions. In terms of practice, the figures inform us that adolescents nowadays do have prosocial involvement experiences, yet prosocial involvement is a long-term activity, and thus the processes relating to the maintenance of the activity need to be considered. Besides, it is essential to have motivated self-initiated and self-endorsed prosocial involvement. In terms of research, further study to examine how individual characteristics, school factors, the nature of activities, and other variables are related to adolescent prosocial involvement.

Past volunteer experience is a crucial factor for prosocial involvement. Activities that are likely to expose adolescents to messages about the importance of altruistic action provide psychological motivation to participate. However, our current local programs focus more on the doing/service part but less on the reflection part. Programs that would help adolescents to reflect on the meaning of altruistic actions, consolidate their experiences, and develop strong civic values and interests in serving others are recommended.

Studies of parenting styles and prosocial involvement inform us that people who experienced parental warmth tended to have a higher level of empathic concern and more prosocial involvement. Therefore, the long-term strategy then, is to have parent education programs and the promotion of prosocial involvement in adolescents going hand in hand. Partnering with Parent-Teacher Associations in schools to develop comprehensive programs for both adolescents and their parents can achieve a better outcome for positive youth development.

There is evidence from overseas that moral reasoning is associated with prosocial involvement in adolescence. Studies indicate that higher levels and stages of moral reasoning and other-oriented modes of moral reasoning are related positively to prosocial involvement [24]. Recent theoretical approaches to the psychology of moral development suggest that both moral emotions and moral motivation serve as important underpinnings of prosocial, morally relevant behaviour. Further study within the local context to enrich our understanding of the influence of moral motivation and moral emotion, and its relation to civic values and prosocial involvement is suggested.

There is consistency in the findings about the development of prosocial dispositions and prosocial personality dispositions that emerge by adolescence and are somewhat stable into adulthood [57]. These findings underline the importance of early intervention. It is essential to foster children with prosocial values and behaviour before entering adolescence. Systematic research to study prosocial dispositions and studies to evaluate program effectiveness are recommended. It would be interesting to look at the relationship between age and prosocial development in the Chinese culture context and to accumulate the findings of research on program evaluation.

In conclusion, humans are genetically predisposed to be prosocial and helpful. With adequate promotion, and in a facilitating context this good character and well-internalized value can be harnessed to achieve positive adolescent development outcomes.
